# Elexacaftor–tezacaftor–ivacaftor enhances first-phase insulin secretion and improves glucose control in cystic fibrosis

**DOI:** 10.1530/EC-25-0690

**Published:** 2026-01-12

**Authors:** Anna Edlund, Ulrika Lindberg, Katarina Fagher, Lisa I Påhlman

**Affiliations:** ^1^ Department of Clinical Sciences Lund, Section for Infection Medicine, Lund University, Lund, Sweden; ^2^ Lung/Allergology, Skåne University Hospital, Lund, Sweden; ^3^ Department of Clinical Sciences, Lund University, Lund, Sweden; ^4^ Department of Endocrinology, Skåne University Hospital, Lund, Sweden; ^5^ Clinic for Infectious Diseases, Skåne University Hospital, Lund, Sweden; ^6^ Wallenberg Centre for Molecular Medicine, Lund University, Lund, Sweden

**Keywords:** cystic fibrosis, CF-related diabetes, CFTR, insulin, glucagon, GLP-1

## Abstract

**Objective:**

Cystic fibrosis (CF) is a congenital condition caused by mutations in the CF transmembrane conductance regulator (*CFTR*) gene. CF-related diabetes (CFRD) is a common comorbidity among people with CF (pwCF) and is associated with increased morbidity. Previous *in vitro* studies have suggested that CFTR dysfunction is involved in the pathogenesis of CFRD. This prospective study aimed to evaluate the role of CFTR in glucose homeostasis by comparing glucose, insulin, C-peptide, glucagon and GLP-1 responses during an oral glucose tolerance test (OGTT) in pwCF and healthy controls and in pwCF before and after initiating elexacaftor–tezacaftor–ivacaftor (ETI) therapy.

**Methods:**

Twenty-four pwCF were enrolled, of which 18 underwent OGTT both before and after ETI initiation. Ten healthy controls were included for comparison. Plasma samples were collected at standard OGTT time points, including an additional 15 min sample. Hormonal and glucose responses during OGTT were compared across groups and in pwCF before and after starting ETI.

**Results:**

Compared with healthy controls, pwCF exhibited impaired glucose regulation, delayed insulin secretion, decreased insulin sensitivity index and reduced disposition index, indicating beta-cell dysfunction. In addition, p-glucagon levels were elevated in pwCF. Following ETI therapy, pwCF showed improved glucose control, increased first-phase insulin secretion and normalized glucagon secretion.

**Conclusions:**

These findings support a role for CFTR in islet hormone regulation, implicating direct effects on beta-cell function and a potential role in suppressing glucagon secretion.

## Introduction

Cystic fibrosis (CF) is a monogenetic disease caused by mutations in the plasma membrane anion-channel CF transmembrane conductance regulator (CFTR). The condition is characterized by persistent airway infections leading to inflammation and progressive lung function decline (1). CF-related diabetes (CFRD) is the most common comorbidity and is associated with an increased morbidity and mortality (2). The incidence increases with age, affecting nearly 20% of adolescents and 50% of adults (3, 4). In addition, various degrees of glucose intolerance are common among pwCF and may precede diabetes (3). Due to the high risk of developing CFRD, guidelines recommend annual screening with oral glucose tolerance tests (OGTTs) starting at the age of 10. CFRD is a unique clinical diagnosis with some clinical features overlapping with both type 1 and type 2 diabetes. CFRD is primarily characterized by insulin deficiency, with minimal or absent insulin resistance (5, 6, 7). The underlying pathophysiology of CFRD is under debate and has recently been reviewed (8). Multiple contributing factors have been proposed. The majority of pwCF are pancreatic insufficient due to the progressive destruction and inflammation in the exocrine pancreas starting already *in utero*. This inflammatory environment is believed to negatively impact the endocrine pancreas. In line with this hypothesis, pro-inflammatory cytokines, oxidative stress and islet extrinsic factors originating from the exocrine pancreas have been implicated in islet dysfunction (9, 10, 11). Moreover, histopathological studies of CF pancreatic tissue have demonstrated that although islet cell mass is largely preserved, islets undergo remodeling and show partial loss of beta-cells (10, 12, 13). However, the occurrence of CFRD in pancreatic-sufficient pwCF suggests that other mechanisms are involved (14). Notably, a direct effect of CFTR in pancreatic islets has been suggested. CFTR expression has been demonstrated in islet alpha- and beta-cells (15, 16, 17, 18, 19, 20, 21), and pharmacological inhibition of CFTR in isolated human islets reduces insulin secretion (15, 17) and enhances glucagon secretion (16). Similar results have been shown in animal models of CF (19, 22, 23, 24).

Insulin is secreted biphasically in response to glucose, with a first rapid phase (15–30 min into the OGTT), driven by the readily released pool of insulin granules, primarily reflecting the beta-cell function (25, 26, 27, 28). The sustained second phase involves both beta-cell intrinsic and extrinsic factors (26, 29). Notably, pwCF exhibit a reduced first-phase insulin response (30), suggesting beta-cell dysfunction.

In the past decade, small molecular modulators that directly target dysfunctional CFTR and improve the overall function of the plasma membrane protein have been introduced. The triple modulator elexacaftor–tezacaftor–ivacaftor (ETI) was approved by the FDA in 2019 for the treatment of pwCF with at least one copy of the most common F508del mutation or selected class II mutations, carried by 80–90% of the CF population. ETI has been shown to improve morbidity and mortality, and there is evidence that ETI improves glycemia in CFRD (31, 32, 33, 34, 35, 36). However, the underlying mechanisms of the effects that ETI might have on glucose homeostasis are not fully understood.

The aim of this study was to evaluate the impact of ETI on insulin secretion and overall glucose homeostasis in individuals with CF. We hypothesized that restoration of CFTR function with ETI would improve glucose control in pwCF via a direct effect on beta-cell function. To achieve this, we first compared glucose tolerance, islet hormone levels and incretin secretion between pwCF and healthy controls and subsequently assessed these parameters in pwCF before and after 1–2 years of ETI therapy.

## Methods

### Study design and patient population

This prospective single-center study was performed at Skane University Hospital, Lund, Sweden. To evaluate the role of CFTR in glucose regulation, the study design aimed to collect plasma samples from pwCF at two consecutive routine OGTT evaluations performed with one to two years a part, before and after start of ETI treatment. Patient enrollment started in August 2021, and sample collection was finalized in July 2024. ETI (Kaftrio©) was approved for the treatment of pwCF in Sweden in December 2022.

PwCF registered at the cystic fibrosis center at Skane University Hospital, Lund, Sweden, were eligible for inclusion in the study. Inclusion criteria were age ≥18 years, diagnosis of CF and participation in the annual OGTT screening program at Skåne University Hospital, Lund, Sweden. Exclusion criteria were inability to understand participation in the study by, for example, dementia, confusion or inability to understand the language, a history of organ transplantation, ongoing ETI treatment and OGTT performed at remote hospital. A diagnosis of CFRD discontinues participation in the annual glucose tolerance screening program. For comparison, 10 normoglycemic controls (non-CF NGT) were enrolled. Exclusion criteria included diagnosis of diabetes or diabetes in first-degree relatives, BMI < 19 or >25, pregnancy, gestational diabetes and any daily medications.

The study was approved by the Swedish Ethical Review Authority (reference no. 2021-01908 with amendments 2022-03370-02 and 2022-07322-02). Written informed consent was obtained from all study participants prior to enrollment.

### Oral glucose tolerance test (OGTT)

After overnight fasting, patients received a venous catheter in the median cubital vein at the Clinical Chemistry Department at Skane University Hospital, Lund, Sweden (pwCF) or at the Department of Clinical Studies at Skane University Hospital, Lund, Sweden (non-CF NGT). Before an oral load of 75 mg glucose was administered, a fasting venous sample was collected. Blood sampling was then performed at 30-, 60-, 90- and 120 min according to clinical routine. For the purpose of the study, an extra blood sample was collected at 15 min to assess the early phase of insulin secretion. All study samples were collected in aprotinin vacutainer tubes (BD Plymouth, UK) to prevent degradation of glucagon. Blood samples were centrifuged at 2,000 *g* for 10 min, and the plasma supernatants were stored in aliquots in −80°C until further analysis.

### Clinical characterization of study participants according to glucose tolerance

Based on the OGTT p-glucose curve, according to current CFRD clinical guidelines (37), normal glucose tolerance (NGT) was defined as fasting p-glucose ≤6.0 mmol/L, 1 h p-glucose ≤11.1 mmol/L and 2 h p-glucose ≤7.8 mmol/L; impaired fasting glucose (IFG) with fp-glucose 6.1–6.9 mmol/L; impaired glucose tolerance (IGT) as fp-glc ≤7.0 and 2 h p-glucose of 7.9–11 mmol/L; and indeterminate glucose tolerance (INDET) as any mid-OGTT p-glucose ≥11.1 mmol/L with a normal fasting and 2 h glucose. CFRD was defined as fp-glucose ≥7.0, confirmed with a second elevated fp-glucose, and/or 2 h glucose ≥11.1 mmol/L. Because of the limited sample size, IFG, INDET, IGT and CFRD were grouped as glucose intolerant (pwCF GI) in this study.

### Glucose measurements

Plasma glucose was analyzed in duplicate using HemoCue Hb201 (Sweden). According to the manufacturer’s instruction, a correction factor 1.11 was used to normalize plasma glucose to whole blood measurements. The results were compared to whole blood glucose concentrations from the standard OGTT time points that were analyzed at the Department of Laboratory Medicine as part of standard clinical care for pwCF. These results were retrieved from the medical journals. There were no significant differences between whole blood glucose measurements and plasma glucose measurements using HemoCue (Supplementary Fig. 1 (see section on Supplementary materials given at the end of the article)).

### Hormonal analysis

Plasma proinsulin was measured using ELISA (Mercodia, Sweden). Insulin, C-peptide and total glucagon-like peptide-1 (GLP-1) were measured using a multiplex U-PLEX, and glucagon was measured using a single U-PLEX from Mesoscale immunoassays (Mesoscale diagnostics LLA, USA). Data were processed using MSD Discovery workbench. For further comparison of analytes, concentration conversion factors were retrieved from MSD company (NIBSC international units). All samples were analyzed in duplicate.

### Estimating beta-cell function and insulin sensitivity

Beta-cell function and insulin sensitivity were estimated from OGTT sampling data as follows:

Glucose excursion was estimated using the incremental area under the curve for glucose (AUC_glucose_). Beta-cell function was assessed by calculating the incremental area under the insulin curve (AUC_insulin_) and the insulinogenic index (IGI), defined as the ratio of the change in plasma insulin to the change in plasma glucose during the first 30 min of the OGTT: (I_30_-I_0_)/(G_30_-G_0_) (38, 39). In addition, we calculated the corresponding IGI for the first 15 min: (I_15_-I_0_)/(G_15_-G_0_).

Insulin resistance was assessed using the homeostatic model assessment of insulin resistance (HOMA-IR) (40) and the Matsuda insulin sensitivity index (ISI_Matsuda_) (39). While HOMA-IR primarily reflects hepatic insulin resistance, the Matsuda index captures whole-body insulin sensitivity. A higher ISI_Matsuda_ indicates greater insulin sensitivity, whereas a higher HOMA-IR indicates increased insulin resistance.

The disposition index, a measurement of beta-cell compensatory capacity in relation to insulin sensitivity, was calculated to assess the adequacy of insulin secretion in the context of insulin resistance, where a lower value indicates impaired beta-cell function (39).

The fold increase of proinsulin was calculated by dividing each proinsulin value to its corresponding fasting value.

### Statistical analysis

Statistical calculations were performed using GraphPad Prism version 10.2.3 (GraphPad software, USA). Comparisons between groups were made using the Kruskal–Wallis test. In case of significant differences (*P* < 0.05), Mann–Whitney U was used for pairwise group comparisons. Categorical variables were compared using Fisher’s exact test. The Wilcoxon matched-pairs signed-rank test was used to calculate paired observations of the ETI effect. A two-tailed *P*-value <0.05 was considered statistically significant.

## Results

### Study population

A total of 24 pwCF were included in the study and underwent a baseline OGTT. Based on p-glucose curves, 42% (*n* = 10) of participants exhibited normal glucose tolerance (CF NGT), while 58% (*n* = 14) were classified as glucose intolerant (CF GI) (Table 1). The CF GI group consisted of one individual with IFG, five with INDET, six with IGT and two participants fulfilling the criteria for CFRD. For comparison, 10 healthy control subjects with normal glucose tolerance (non-CF NGT) underwent OGTT using the same protocol as the pwCF cohort. Baseline characteristics for all study participants are shown in Table 1. Of the initial 24 pwCF, 18 were re-evaluated with OGTT sampling after one year of ETI therapy (clinical characteristics are presented in Table 2). Among the six participants who did not undergo a second OGTT, one had been referred to another CF center, one had not initiated ETI therapy, and four did not complete the second OGTT within the study timeframe.

**Table 1:** Baseline characteristics for pwCF and healthy controls (non-CF NGT).

	CF	Non-CF NGT
	*n* = 24	*n* = 10
Age (years)	32 (25–39)	23.5 (22–26)
Male gender; *n* (%)	15 (63)	5 (50)
Pancreatic insufficiency; *n* (%)	24 (100)	0 (0%)
FEV1pp	77 (56–87)	NA
BMI	23.0 (21.4–24.0)	23.4 (21.3–24.4)
HbA1c (mmol/mol)	40 (36–42)	NA
Lumacaftor/ivacaftor; *n* (%)	9 (38%)	0 (0%)
Normal glucose tolerance; *n* (%)	10 (42%)	10 (100%)
Glucose intolerance; *n* (%)	14 (58%)	0 (0%)

FEV1pp, forced expiratory volume in 1 s in percent of predicted.

BMI, body mass index.

Data are presented as median and interquartile range unless otherwise stated.

**Table 2:** Characteristics of the pwCF cohort before and after the start of ETI.

	Before ETI	After ETI	*P*-value
	*n* = 18	*n* = 18	
Age (years)	32 (26–42)	34 (28–43)	<0.001
Male gender; *n* (%)	12 (67)	12 (67)	
FEV1pp	74 (54.5–84)	83.5 (65–95)	<0.001
BMI	22.7 (21.4–24.1)	23.6 (21.8–25.1)	0.02
HbA1c mmol/mol	41 (37–44)	37 (35–40)	<0.001
Lumacaftor/ivacaftor; *n* (%)	6 (33)	0 (0)	0.02
Normal glucose tolerance; *n* (%)	6 (33)	8 (44)	0.49
Glucose intolerance; *n* (%)	12 (67)	10 (56)	0.49
INDET/IFG; *n*	6	5	
IGT; *n*	5	3	
CFRD; *n*	1	2	

FEV1pp, forced expiratory volume in 1 s in percent of predicted.

BMI, body mass index.

INDET, indeterminate glucose tolerance.

IFG, impaired fasting glucose.

IGT, impaired glucose tolerance.

CFRD, CF-related diabetes.

Data are presented as median and interquartile range unless otherwise stated.

### Glucose regulation in pwCF compared to non-CF controls

**Figure 1:**
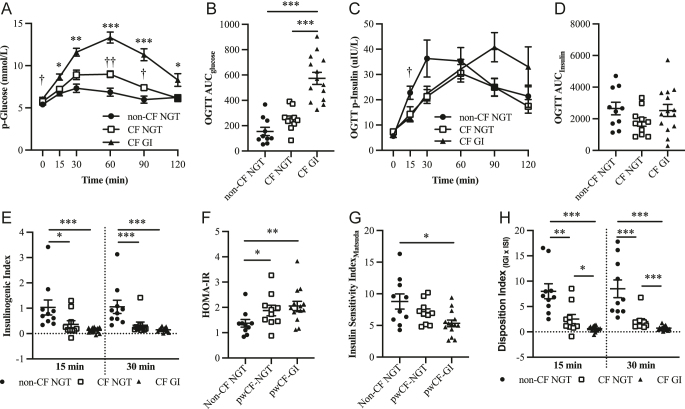
Glucose regulation during OGTT in healthy controls with normal glucose tolerance (non-CF NGT, *n* = 10, open squares), pwCF with normal glucose tolerance (pwCF NGT, *n* = 9, black dots) and pwCF with glucose intolerance (pwCF GI, *n* = 15, black triangles). (A) p-glucose curves during OGTT. (B) Glucose incremental area under the curve (AUC_glucose_). (C) p-insulin curves during the OGTT. (D) Insulin incremental area under the curve (AUC_insulin_) during OGTT. (E) Insulinogenic index (IGI) measured after 15 min (left section of the graph) and after 30 min (right side of the graph). (F) HOMA-IR. (G) Whole-body insulin resistance estimated using the insulin sensitivity index by Matsuda. (H) Disposition index estimated at 15 min (left half of the graph) and after 30 min (right side of the graph). Data are presented as mean ± SEM. ^†^*P* < 0.05 non-CF vs CF NGT, ^††^*P* < 0.01 non-CF vs CF NGT, ^†††^*P* < 0.001 non-CF vs CF NGT. *P < 0.05, **P < 0.01, ***P < 0.001 CF NGT vs CF GI.

Comparisons of p-glucose levels during the OGTT demonstrated that pwCF had a right-shifted glucose curve independent of glucose tolerance, with a delayed p-glucose peak in both pwCF NGT and pwCF GI compared to non-CF NGT (Fig. 1A). AUC_glucose_ did not differ significantly between non-CF NGT and pwCF NGT but was increased in pwCF GI (Fig. 1B). Insulin secretion peaked after 30 min in non-CF NGT and then reached a plateau phase followed by a slope back to fasting values after 120 min (Fig. 1C). Although glucose tolerance was normal, the pwCF NGT group exhibited a delayed insulin peak at 60 min, which was further delayed to 90 min in the pwCF GI cohort. In the latter group, p-insulin did not return to baseline fasting values by the end of the OGTT. AUC_insulin_ did not differ significantly between the groups (Fig. 1D). To further investigate beta-cell function, insulinogenic index (IGI) was calculated at both 15 and 30 min time points in the OGTT (Fig. 1E). We found that IGI was significantly decreased in pwCF NGT at both 15 min (*P* < 0.05) and 30 min (*P* < 0.001) compared to non-CF NGT. Insulin resistance measured by HOMA-IR was normal in the non-CF NGT group but significantly increased in both pwCF-NGT (*P* < 0.05) and pwCF GI (*P* < 0.01) (Fig. 1F). However, when we calculated whole-body insulin resistance using the insulin sensitivity index developed by Matsuda (ISI_Matsuda_), we found reduced insulin sensitivity in pwCF GI, but not in pwCF NGT, compared with non-CF NGT (Fig. 1G). The disposition index, a measurement of beta-cell compensatory capacity in relation to insulin sensitivity, was significantly lower in pwCF NGT at both 15 min (*P* < 0.01) and 30 min (*P* < 0.001) compared to non-CF NGT (Fig. 1H). The disposition index was further reduced in pwCF GI at both time points (*P* < 0.001 and *P* < 0.001, respectively) compared to non-CF NGT. These findings indicate reduced beta-cell function in the pwCF cohort compared to non-CF controls.

### Hormonal regulation in pwCF compared to non-CF controls

**Figure 2:**
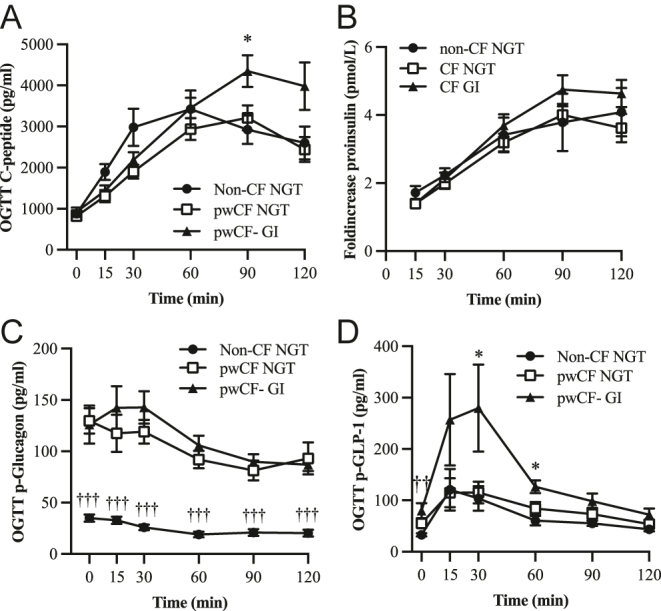
Islet hormonal regulation in healthy controls with normal glucose tolerance (non-CF NGT, *n* = 10, black dots), pwCF with normal glucose tolerance (pwCF NGT, *n* = 9, open squares) and pwCF with glucose intolerance (pwCF GI, *n* = 15, black triangles) during OGTT. (A) Plasma C-peptide during the OGTT. (B) Plasma proinsulin presented as the fold increase of fasting levels of p-proinsulin. (C) Plasma glucagon curves during the OGTT. (D) Plasma glucagon-like peptide-1 (GLP-1) curves during the OGTT. Data are presented as mean ± SEM. ^††^*P* < 0.01, ^†††^*P* < 0.001 non-CF vs CF NGT. *P < 0.05 CF NGT vs CF GI.

Plasma C-peptide curves during the OGTT mirrored the insulin curves showing a delayed peak in pwCF NGT and pwCF GI compared to non-CF NGT (Fig. 2A). However, C-peptide AUCs were comparable between groups (Supplementary Fig. 2A). Proinsulin curves during OGTT were also similar between cohorts (Fig. 2B).

As shown in Fig. 2C, fasting plasma glucagon levels were significantly elevated in pwCF, regardless of glucose tolerance status. In the pwCF GI group, glucose appeared to fail to rapidly suppress plasma glucagon levels. In addition, the AUC for glucagon was significantly increased in both pwCF NGT (*P* < 0.001) and pwCF GI (*P* < 0.001) compared to non-CF controls (Supplementary Fig. 2B).

Plasma GLP-1 was significantly increased in pwCF GI compared to pwCF NGT (Fig. 2D). The AUCs for GLP-1 were increased in both pwCF NGT (*P* < 0.01) and pwCF GI (*P* < 0.01) compared to non-CF NGT (Supplementary Fig. 2C).

### Longitudinal changes in glucose regulation after initiating ETI

To investigate the role of CFTR in glucose regulation *in vivo*, we compared baseline OGTT data (OGTT_1_) with results from a follow-up OGTT conducted after initiating CFTR-modulator therapy with ETI (OGTT_ETI_). During the timeframe of the study, 18 pwCF underwent a follow-up OGTT after treatment with ETI for a median of 13 months (range 10–23 months). Before starting ETI treatment, 6 pwCF were classified as NGT and 12 were glucose intolerant (5 INDET, 1 IFG, 5 IGT and 1 CFRD). On ETI, the number of NGT increased to eight. Four participants with INDET or IFG improved to normal glucose tolerance after the start of ETI, whereas two NGT developed INDET and IGT, respectively. One individual with IGT at baseline progressed to CFRD despite ETI therapy (Table 2 and Supplementary Fig. 3).

**Figure 3:**
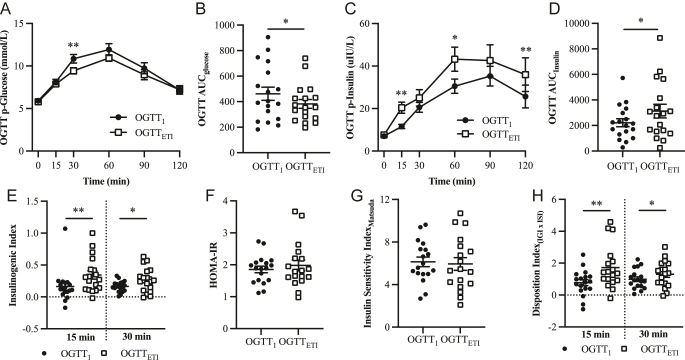
Glucose regulation during an oral glucose tolerance test (OGTT) before (OGTT_1_, black circles, *n* = 18) and after treatment with elexacaftor–tezacaftor–ivacaftor (OGTT_ETI_, open squares, *n* = 18). (A) OGTT p-glucose curve. (B) OGTT p-glucose incremental area under the curve (AUC_glucose_). (C) Plasma insulin curve during OGTT. (D) OGTT insulin incremental AUC (AUC_insulin_). (E) Insulinogenic index at 15 min (left part of the graph) and at 30 min (right part of the graph). (F) HOMA-IR and (G) whole-body insulin resistance index estimated using the insulin sensitivity index by Matsuda. (H) Disposition index (disposition index_(IGIxISI)_) at 15 min (right side of the graph) and at 30 min (left side of the graph), as indicated. Data are presented as mean ± SEM. *P < 0.05, **P < 0.01.

Comparisons of p-glucose curves during OGTT before and after the start of ETI demonstrated significantly lower p-glucose levels at the 30 min time point (Fig. 3A). Improvement in glucose tolerance by ETI was further illustrated by a significant decrease in AUC_glucose_ (*P* < 0.05; Fig. 3B). When plasma insulin was analyzed, we found a significant increase at 15 min in OGTT_ETI_ compared to OGTT_1_ (*P* < 0.01; Fig. 3C). Similarly, AUC_insulin_ increased significantly after ETI treatment (Fig. 3D). To further investigate the effect of ETI at the beta-cell level, insulinogenic index was calculated. As shown in Fig. 3E, ETI treatment improved insulinogenic index at both 15 min (*P* < 0.01) and 30 min (*P* < 0.05). Insulin resistance, both in the liver calculated with HOMA-IR and of whole-body using Matsuda insulin sensitivity index, was unaffected by ETI (Fig. 3F and G). ETI significantly increased disposition index at both 15 min (*P* < 0.01) and 30 min (*P* < 0.05; Fig. 3H), which further suggests that ETI positively affects the beta-cell.

### Effect on hormone secretion after initiating ETI

**Figure 4:**
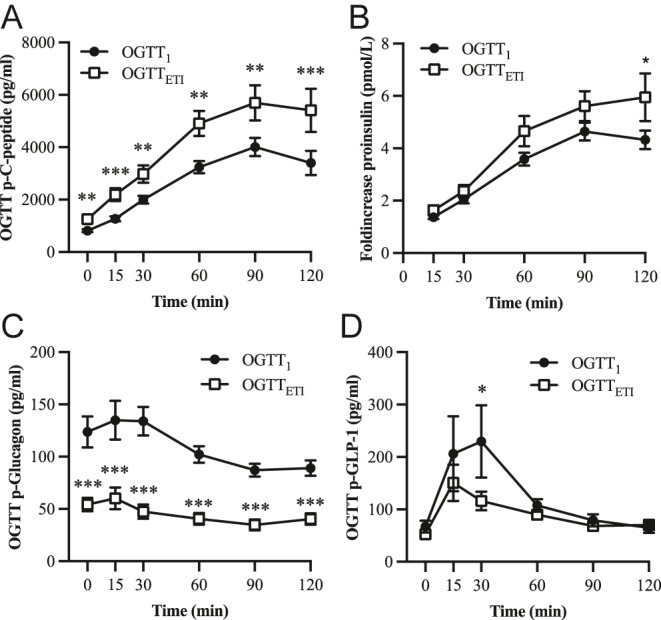
Glucose-regulated hormones during OGTT performed before (OGTT_1_, black dots, *n* = 18) and after the start of elexacaftor–tezacaftor–ivacaftor treatment (OGTT_ETI_, open squares). (A) OGTT plasma C-peptide curve. (B) Fold increase of plasma proinsulin during OGTT. (C) OGTT plasma glucagon curves and (D) plasma GLP-1 curves. Data are presented as mean ± SEM. *P < 0.05, **P < 0.01, ***P < 0.001.

The OGTT c-peptide curve was significantly improved at all time points after treatment with ETI (Fig. 4A), and the AUC_c-peptide_ was significantly increased (*P* < 0.01) compared to before the start of ETI (Supplementary Fig. 4A). The fold increase of p-proinsulin was significantly higher at the 120 min time point with ETI treatment (Fig. 4B).

The overall OGTT p-glucagon curve improved after the start of ETI treatment and nearly restored glucose-induced suppression of glucagon (Fig. 4C). Consequently, AUC_glucagon_ was significantly reduced (*P* < 0.001, Supplementary Fig. 4B). Total GLP-1 secretion was reduced by ETI and peaked earlier (15 vs 30 min; Fig. 4D). Thus, ETI treatment resulted in GLP-1 OGTT kinetics similar to the healthy control group (see Fig. 2D). The reduced GLP-1 secretion in response to ETI treatment was further supported by a decreased OGTT GLP-1 AUC (*P* < 0.01; Supplementary Fig. 4C).

## Discussion

In this study, we analyzed glucose regulation during OGTT in pwCF with normal or impaired glucose tolerance and compared them to healthy controls. To further evaluate the role of CFTR in glucose homeostasis, we also assessed OGTT responses in pwCF before and during treatment with the CFTR modulator ETI. Our findings demonstrate that even pwCF with normal glucose tolerance exhibit dysfunctional glucose handling, characterized by a delayed glucose peak that became more pronounced with worsening glucose intolerance (Fig. 1A). Insulin secretion was significantly reduced in pwCF compared to healthy controls at 15 min (*P* < 0.01) and 30 min (*P* < 0.05), corresponding to impaired first-phase insulin secretion. These findings are consistent with previous studies showing an impaired insulin response in pwCF during OGTT, irrespective of glucose tolerance status (14). In addition, the insulin peak during OGTT was delayed in pwCF NGT and further delayed in pwCF GI (Figs 1C and 2A), suggesting that glucose intolerance in CF is mainly driven by a right-shifted insulin response. This pattern reflects reduced first-phase insulin secretion and progressive beta-cell dysfunction, rather than overt insulin deficiency. Insulin is secreted in a biphasic manner with a rapid phase, generally termed the first-phase insulin secretion, lasting up to 30 min, and a slower sustained second-phase insulin secretion. The first-phase insulin secretion is mainly dependent on the beta-cell function, while the second phase depends on additional beta-cell extrinsic factors. Indeed, analyses of insulinogenic index at 15 and 30 min (Fig. 1E) demonstrated impaired beta-cell function in the pwCF cohort. In CF, insulin resistance has not been implicated to be a driving force on glucose intolerance and ultimately CFRD (7, 41). Here, we show that pwCF has an increased insulin resistance in the liver, whereas whole-body insulin resistance was similar to non-CF NGT. Indeed, when calculating the disposition index, which is considered as an estimate of the beta-cells’ ability to compensate for insulin resistance, it was clearly reduced in pwCF, indicating a defect at the beta-cell level.

Previous *in vitro* studies have suggested that the dysregulated glucose metabolism observed in CF is caused by CFTR dysfunction resulting in an impaired early-phase insulin release from beta-cells (15, 22). In the present study, we demonstrate that overall glucose control improves in pwCF after the start of the CFTR-modulator therapy ETI (Fig. 3A). Moreover, p-insulin increased significantly at 15 min during OGTT, corresponding to the first-phase insulin secretion (Fig. 3C). Similarly, insulinogenic index increased after the start of ETI (Fig. 3E), indicative of improved beta-cell function. Further strengthening an improved beta-cell function by ETI is the increased disposition index at both 15- and 30 min (Fig. 3H). Taken together, our data suggest that CFTR is important for an effective insulin secretion and glucose control. Importantly however, although ETI therapy resulted in an improved glucose control at the group level, some individuals deteriorated despite ETI treatment (Supplementary Fig. 3). These findings suggest that ETI does not protect from progression toward CFRD and that continuous OGTT screening is still important in CF care.

OGTT plasma glucagon levels were significantly higher in pwCF, regardless of glucose tolerance (Fig. 2C). Moreover, in pwCF GI, the glucagon secretion continued to increase despite an increase in plasma glucose during the first 30 min of the OGTT. Furthermore, ETI treatment had a profound effect on plasma glucagon by reducing fasting glucagon secretion as well as throughout the OGTT (Fig. 4C). These data support previously published *in vitro* data demonstrating CFTR expression in human alpha cells and that pharmacological inhibition or loss-of-function mutations contribute to impaired suppression of glucagon secretion during a glucose load (16, 20). The incretin GLP-1 is released from intestinal L-cells in response to nutrient intake and amplifies glucose-stimulated insulin secretion (42). GLP-1 acts on the pancreatic beta-cell by increasing intracellular cAMP and ATP levels (42), which is also required for CFTR activation. Interestingly, pwCF had elevated fasting GLP-1 levels, and OGTT-stimulated plasma GLP-1 was significantly higher in pwCF GI and remained elevated during the total timeframe of the OGTT. Despite the rapid increase in plasma GLP-1 in pwCF GI, a corresponding increase in insulin secretion was absent due to a reduced first-phase insulin response. Moreover, GLP-1 levels decreased following initiation of ETI therapy. These findings raise the possibility of GLP-1 resistance in pwCF GI. Supporting this hypothesis, reduced GLP-1 receptor expression has been reported in CFRD (43). Alternatively, improved glycemic control following ETI therapy may attenuate the stimulus for GLP-1 secretion, suggesting that the post-ETI reduction in GLP-1 represents a normalization of the incretin response secondary to improved glucose handling. However, this study assessed total GLP-1 levels rather than bioactive GLP-1, and further research is needed to determine whether similar trends are observed for the active form.

This study offers several strengths that contribute to our understanding of glucose regulation in pwCF. Although several studies have reported altered glucose regulation in pwCF (5, 14), we compare pwCF with healthy control subjects within the same study. This comparison provides a clearer assessment of the metabolic alterations associated with CF. In addition, the inclusion of OGTT data both before and after the initiation of ETI therapy enabled us to evaluate how restoration of CFTR function affects glucose homeostasis and insulin secretion dynamics. Another key strength is the inclusion of the 15 min time point during OGTT, which is not part of the standard OGTT protocol. This addition allowed us to capture the early phase of insulin secretion – an important aspect for the understanding of the role of CFTR in glucose regulation.

Nevertheless, our study has several limitations that should be taken into consideration. Most importantly, the improved glycemic control following ETI therapy may involve additional mechanisms beyond a direct effect on CFTR within pancreatic beta-cells. For instance, ETI may reduce systemic and intra-pancreatic inflammation, indirectly influencing glucose homeostasis. Moreover, this was a single-center study with a relatively small sample size, which limits the generalizability of the findings. The limited number of patients also precludes the possibility of conducting subgroup analyses on individuals with CFRD, IGT, INDET, etc. Furthermore, since the OGTT screening program ceases when patients are diagnosed with CFRD, relatively few patients with CFRD were included in our study population. This may have restricted our ability to draw conclusions on glucose regulation in specific subgroups. Despite these limitations, our findings identify significant differences in the hormonal regulation of plasma glucose levels between pwCF and healthy controls, as well as notable changes following CFTR modulation with ETI therapy.

In conclusion, our findings indicate that pwCF exhibit impaired glucose regulation and beta-cell function compared to healthy controls. Treatment with the CFTR-modulator ETI enhanced glucose control and insulin secretion in pwCF, suggesting a pivotal role of CFTR in the early phase of insulin release from beta-cells.

## Supplementary materials



## Declaration of interest

UL received honoraria from Vertex and AstraZeneca.

## Funding

This study was funded by the Swedish Heart and Lung Foundation (grant F2022/2227), the Alfred Österlund Foundation (grant F2022/190), the Swedish Cystic Fibrosis Association (grant F2022/625), the Swedish government funds for clinical research (ALF, grant 40204), the Swedish Research Council (grant 81248), and the Knut and Alice Wallenberg Foundation, Medical Faculty at Lund University and Region Skåne (grant 81234).

## Author contribution statement

AE planned the study, researched data and wrote the manuscript. UL contributed to study design and discussion. KF contributed to study design and discussion and edited the manuscript. LIP planned and supervised the study and wrote the manuscript. All authors read and approved the manuscript. LIP and AE are the guarantors of this work and take responsibility for the integrity of the data and the accuracy of the data analysis.
