# Supplementary material for: Elexacaftor–tezacaftor–ivacaftor enhances first-phase insulin secretion and improves glucose control in cystic fibrosis
Source: Endocr Connect. 2026 Jan 12;15(1):e250690. doi: 10.1530/EC-25-0690 (PMC12809108; doi:10.1530/EC-25-0690)
Supplement: Supplementary file 1 [file supplementary_materials.pdf]

Supplemental figures

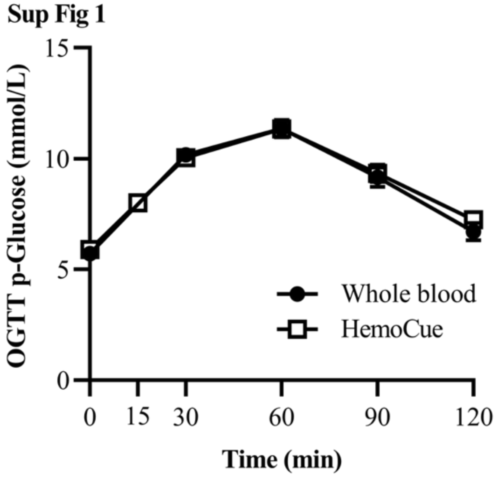

Supplementary Figure 1. Comparison of the plasma glucose curves during the OGTT in whole blood measured in connection with the visit (black dots) and plasma glucose measured in house using Hemocue (open square). The correction factor was added to the Hemocue results as instructed by the manufacture. There was no significant difference between methods, on avarage 3.0 ± 0.6 % variation (n=252 samples measured in duplicates). Data presented as mean ± SEM.

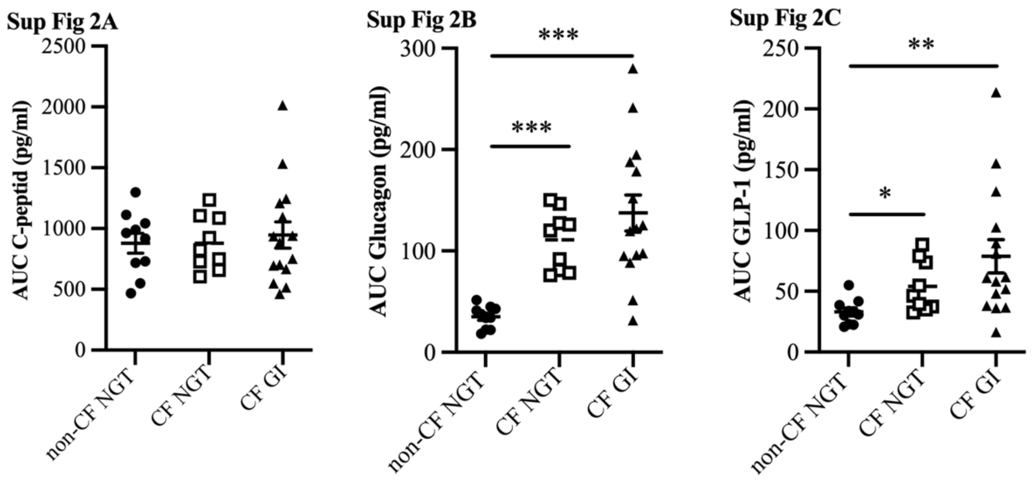

Supplementary figure 2. OGTT incremental area under the curve (AUC) for the glucose regulated hormones. In **(A)** C-peptide, **(B)** glucagon and **(C)** Glucagon-like peptide-1 (GLP-1). Data presented as mean±SEM. *p<0.05, **p<0.01, ***p<0.001.

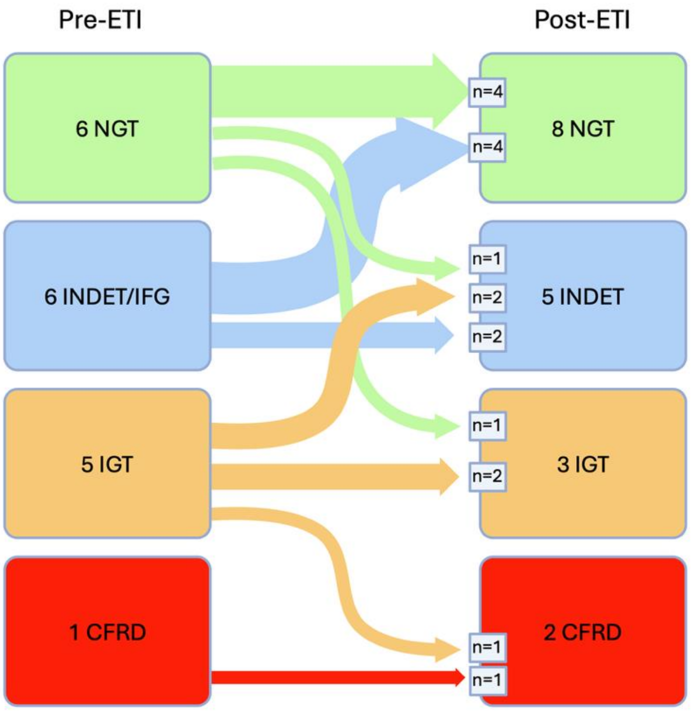

Supplementary figure 3. Classification of glucose tolerance in pwCF (n=18) before and after initiation of ETI therapy. NGT: normal glucose tolerance, INDET: Indeterminate glucose tolerance, IFG: Impaired fasting glucose, IGT: Impaired glucose tolerance, and CFRD: CF-related diabetes.

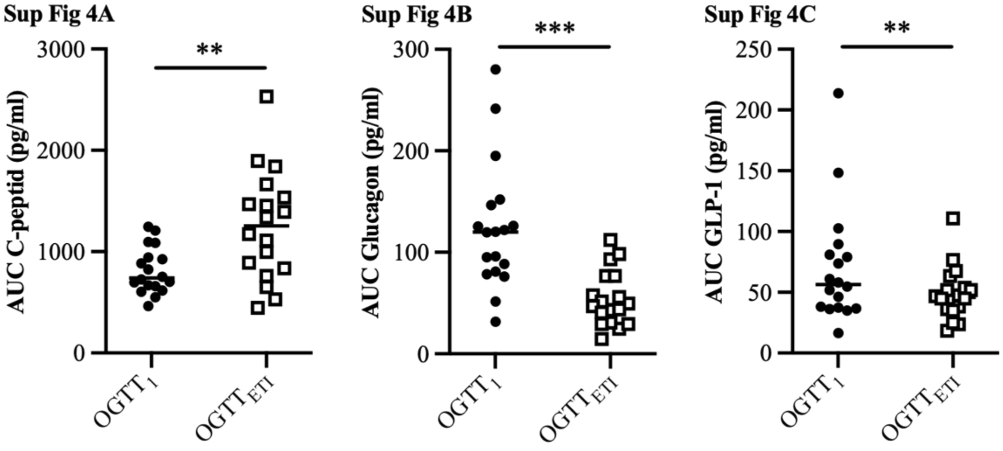

Supplementary figure 4. OGTT incremental area under the curve (AUC) quantified for the glucose regulated hormones before ($OGTT_1$) and on treatment with Elezacaftor-Tezacaftor-Ivakaftor ($OGTT_{ETI}$). In **(A)** C-peptide , **(B)** Glucagon and in **(C)** **G**lucagon-like protein-1 (GLP-1). Data presented as mean±SEM. **$p<0.01$, ***$p<0.001$ $OGTT_1$ vs $OGTT_{ETI}$.
